# Nervous system disorders across the life course in resource-limited settings

**DOI:** 10.1038/nature16031

**Published:** 2015-11-19

**Authors:** Gretchen L. Birbeck, Ana-Claire Meyer, Adesola Ogunniyi

**Affiliations:** 1 Epilepsy Division, Department of Neurology, University of Rochester, 265 Crittenden Boulevard, CU420694, Rochester, New York 14642-0694, USA; 2 Chikankata Epilepsy Care Team, Chikankata Hospital, Private Bag S2, Mazabuka, Zambia; 3 Department of Neurology, Yale University, P.O. Box 208018, New Haven, Connecticut, 06520-8018, USA; 4 Kenya Medical Research Institute, Box 614-40100, Kisumu, Kenya; 5 Department of Medicine, University of Ibadan, PMB 5016, Ibadan 200001, Nigeria

## Abstract

The resiliency of the adult nervous system is markedly affected by the environment and the circumstances during infant and child development. As such, adults in resource-limited settings who may have experienced early deprivation are particularly vulnerable to subsequent neurological disorders. Adult populations in countries with relatively recent advances in economic development may still have a higher susceptibility to neurological illness or injury that is reflective of the socioeconomic environment that was present during that population’s infancy and childhood. Brain and peripheral nervous system research conducted over the past decade in resource-limited settings has led to an impressive and growing body of knowledge that informs our understanding of neurological function and dysfunction, independent of geography. Neurological conditions feature prominently in the burgeoning epidemic of non-communicable diseases facing low- and middle-income countries. Neurological research in these countries is needed to address this burden of disease. Although the burden of more prevalent and severe neurological disease poses public health and clinical challenges in settings with limited neurological expertise, the same factors, along with genetic heterogeneity and the relative absence of ingrained clinical care practices, offer circumstances well-suited for the conduct of crucial future research that is globally relevant.

Neurological disorders represent a significant proportion of the burden of disease among adults in low- and middle-income countries (LMICs). Stroke, epilepsy and dementia rank among the highest causes of death and disability, and often affect working-age adults. High rates of premature death and disability undoubtedly constrain societies that are striving to advance human development through improvements in health and a more stable economy. Research aimed at elucidating the pathophysiology and epidemiology of adult nervous system disorders across a broad spectrum of settings has advanced our capacity to develop interventions at the population and individual level. Insights gained from developed countries have provided remarkable evidence that deprivation in early life imparts additional health-related vulnerabilities that extend into adulthood^1^. This added susceptibility is an important and generally unmeasured factor that could account for the poor health status among LMICs adults. The burden of non-communicable diseases continues to escalate in LMICs that are still struggling with infectious diseases and maternal health challenges; the need to identify opportunities for optimizing health in older populations is increasingly urgent. In this Review, we detail an overview of some of the insights gained from research in diverse academic, laboratory and community settings and consider what knowledge can be gained from investments in future endeavours.

## EARLY EXPOSURE AND THE ADULT NERVOUS SYSTEM

Neurological disorders of infancy, childhood and adolescence are discussed elsewhere in this collection (see page S161), but if survived, neurological injury in early life may result in adult disability, vulnerability to disease and premature mortality. Therefore, it is crucial to integrate a lifespan approach as we examine what we currently know, as well as research priorities for the future. Central nervous system injuries, infections and infestations during childhood that result in epilepsy, behavioural disorders and intellectual disabilities lay the foundation for a compromised adulthood. Furthermore, the life course and exposures of childhood determine adult neurological health, with childhood deprivation and adolescent injury among the primary risk factors for adult neurological disorders in LMICs. For example, childhood risk factors for epilepsy in sub-Saharan Africa include labour and delivery complications and lower maternal education levels^2^. Data from 1921 and 1936 birth cohorts in Aberdeen, UK, suggest that early nutritional deprivation predisposes people to dementia in later life^3^. Rheumatic heart disease in childhood, which is largely a result of poor access to health care and services, is a common cause of stroke among young adults in Southeast Asia and Africa^4,5^.

Although there is a well-established association between poverty and vulnerability to neurological disorders^6^, shockingly little is known regarding the pathophysiological processes that drive such links — even for exposures as ubiquitous as childhood malnutrition^7,8^. Given the substantial effect that childhood health and the environment (in the broadest sense) have on adult neurological health, longitudinal population-based studies of countries that have (and have not) met key United Nation’s Millennial Development Goals are war-ranted. In the future, these experiments in poverty reduction over relatively short time periods may also offer opportunities to formally evaluate well-developed theories regarding epigenetic responses to environmental change and the fetal origins of adult neurological conditions, particularly life-long cognitive impairment, and later onset dementia^9^.

Although malnutrition remains the principle nutritional challenge in child health in low-income countries, childhood obesity is reaching epic proportions in middle-income regions. Prevalence rates in South Africa (15%), Mexico (25%) and Brazil (23%), reflect the availability of relatively cheap, high-energy foods and increasingly sedentary lifestyles. The ongoing childhood obesity epidemic has resulted in unprecedented prevalence rates of hypertension among children and adolescents^10^. High body mass index, hypercholesterolemia and hypertension in childhood are associated with higher carotid artery intima-medial thickness in young adulthood — an established risk factor of cardiovascular disease and stroke^11^. Moreover, childhood hypertension is associated with hypertension as an adult^12^. Other adult cardiovascular risk factors include those that occur early in life with long-term consequences. Passive tobacco exposure in childhood causes lower high-density lipoprotein cholesterol^13^ and childhood smoking is associated with greater carotid intima-medial thickness as an adult, even if the smoking habit ends before maturity^14^. Although exposure to established stroke risk factors from childhood undoubtedly increases an individual’s overall risk of adult cerebrovascular disease, longitudinal studies are needed to quantify the impact of today’s childhood obesity problem on the adult population of tomorrow.

## LATER LIFE DISEASE AND ADVANCES IN NEUROSCIENCE

Although risk factors for adult neurological disorders can occur in isolation in childhood, if problems such as obesity, tobacco use and hypertension begin in childhood or adolescence they often continue into adulthood. Cardiovascular risk factors and illiteracy play a major part in the growing burden of dementia in LMICs^15^. Without successful programmes aimed at poverty reduction and population-based interventions that curb harmful behaviours and encourage healthier lifestyles, the global prevalence of neurological disorders is likely to increase substantially.

The growing body of neurological research from LMICs has contributed substantially to, and at times transformed, our understanding of numerous neurological disorders, or the pathophysiological processes surrounding them. These contributions include observational^16^ and epidemiological^15^ studies, health-services research^17^, and clinical trials^18^, as well as laboratory-based sciences^19^. Many of these advances address neurological disorders that are common in both developed and less-developed settings^20^. Other contributions are focused on disorders more limited in geographical scope^21^, but these too offer advances on which future research can build.

### Advances in neuroscience basic sciences

The mysteries of neurodegeneration are being revealed through research collaborations between US investigators and LMIC scientists. Researchers from the University of Antioquia in Colombia have shown that viral vectors for RNA interference of cyclin-dependent kinase 5 can reduce neurofibrillary tangles in transgenic Alzheimer’s disease mice^22^, and ongoing investigations are aimed at identifying human genes that modify the age at onset of Alzheimer’s^23^. Researchers at New York University and the University of Uruguay are using salmonella-based Aβ to develop a vaccine for Alzheimer’s^24^. Argentinian investigators are also using recombinant adenoviral vectors and have shown that insulin-like growth factor (IGF) I gene therapy can restore hypothalamic dopaminergic function in senile rats^25^, and that IGF also improves motor function^26^. Collaborative studies often between researchers from multiple countries are contributing to our understanding of the genetic mechanisms of Parkinson’s disease and stroke^27^. Further research of this nature is ongoing through the National Institutes of Health’s H3Africa Initiative with a particular focus on stroke genomics in Africa.

#### Epidemiological studies

Epidemiological studies are well represented among those that contribute to our understanding of the global burden of nervous system disorders. Often initial studies offer more questions than answers, meaning subsequent investigations are required. For example, previous epidemiological estimates from developing regions persistently illustrated a ‘gap’ in epilepsy prevalence versus the lifetime prevalence rates, indicating that either people with epilepsy in developing countries have a higher remission rate or that they have higher rates of premature mortality than their counterparts in wealthier settings. As part of the International League against Epilepsy’s China demonstration project, a prospective study of people with epilepsy in rural China demonstrated high rates of prime adult, seizure-related mortality despite readily accessible treatment^28^, indicating that this gap is from mortality and not disease remission. Multinational collaborations currently underway are elucidating the neurotoxicity of pesticides on both passively exposed children and adult agricultural workers^29^.

Although combined antiretroviral therapy is facilitating the transition of HIV from a fatal disease to a chronic condition, neurological disability remains a significant problem for people with HIV worldwide^30^. HIV-associated neurocognitive disorders (HANDs) are evident in almost half of those with HIV in the United States^31^. An international group working on the clade-specific effects of HIV on the central nervous system has gained important insights into the pathophysiology and prevention of HANDs. Studies in Africa and Latin America identified that HIV-associated neuropathy prevalence rates in those countries are substantially greater than in similar US populations, and point towards a central role for nutritional factors^32^. The studies clearly illustrate the need for further investigations aimed at directing future interventions. Interventions to optimize the cognitive outcomes for people with HIV in regions heavily affected by the disease will be needed to further limit the impact of HIV on human resources in LMICs.

#### Implementation science

Research across the life course is needed for neurological disorders, but translating the knowledge from research into action requires evidence-based approaches to implementation that are locally relevant. For example, researchers studying food-related neurotoxicity have successfully identified safer, feasible food preparatory measures, which have been taken up by communities^33^. Epilepsy is one of the commonest chronic neurological conditions in all settings regardless of economic status. The epilepsy community has called for improved screening and integrated care for the common psychiatric co-morbidities that often devastate people with epilepsy. Barriers to developmental and interventional studies that would facilitate this undertaking include time constraints, limited reimbursement and understaffing^34^. Epilepsy researchers in Zambia collaborating with faculty at the country’s only psychiatric hospital have developed and validated a screening instrument for anxiety and depression that is feasible in their setting and appropriate for the people seeking care^17^. Prior to its implementation, less than 1% of people receiving epilepsy care were treated or referred for psychiatric care. After implementation, this number increased to 32% (ref. 17). Despite the recognition that the psychiatric and social morbidity of epilepsy contributes substantially to the burden of those affected, a recent systemic review found that few rigorous intervention studies aimed at decreasing this social morbidity have been conducted^35^. Local peer support groups in Zambia have been shown to decrease epilepsy-associated stigma and improve medication adherence among young people^36^. This work was accomplished in an environment with time, resource and staffing constraints. The approaches taken may offer guidance to researchers from higher income regions.

#### Diagnostic advances

Rigorous clinical investigations require the development or adaptation, and validation of instruments for categorizing and quantifying disease and disability. One of the chief barriers to research in LMICs is the availability of such instruments. In the past decade LMIC-based researchers have undertaken instrument development for neuropsychiatric disorders and dementia in China; neurodisabilities in Africa^37^; HIV-associated dementia and neuropathies in Kenya^38^ and Zambia^39^; and stroke in Vietnam^40^, Argentina^41^, Portugal^42^ and Nigeria^43^. Methodology and software have been developed to facilitate the use of neuroimaging to characterize and quantitatively describe novel conditions^44^ that could prove important in this era of emerging infectious diseases.

#### Clinical trials

Although clinical trials conducted in LMICs might seem to be the study design that is least likely to inform care in high-income regions, a clinical trial addressing the use of invasive intracranial pressure monitoring conducted in Brazil and Ecuador has forced neurosurgeons and neurointensivists to reconsider long-held ideas of the value of such assessments^45^. Conducting clinical trials in settings that are less burdened by ingrained practice patterns and training conventions may offer crucial opportunities to collect the data that will allow us to examine whether common practices established in the absence of evidence are really warranted.

## OLDER ADULTS TO OLDEST OLD

Frailty and functional disability often afflict old age. Degenerative diseases that affect vision and hearing compound health problems in older people. But these are not inevitable characteristics of ageing. Neurological diseases of old age, for which substantial literature exists in LMICs, include stroke, dementia, Parkinson’s disease and epilepsy. The quality of life of those affected is often poor and major depression may occur^46^.

Stroke is the most important cause of disability and a leading cause of death globally. A third of stroke patients in resource-limited settings die and another third are left with residual disability. The toll on economic productivity in these low-income countries that are poorly equipped to care for these individuals is considerable. According to the World Health Organization, stroke accounts for 55% of the disability-adjusted life years (DALYs) for neurological disorders, whereas Alzheimer’s disease is responsible for 12.0% (ref. 47). Currently, stroke is the most important cause of hospital admission in many African countries and this has been adduced to the high frequency of untreated hypertension. Late presentation in hospitals, poor facilities for adequate care provision and prevention of complications contribute to the high mortality rate. The use of thrombolytic agents is limited because of exorbitant cost and late presentation in hospitals. Data on the risk factors for stroke have come from the INTERSTROKE study, which included participants from five countries in sub-Saharan Africa (Mozambique, Nigeria, South Africa, Sudan and Uganda). The frequency of intracerebral haemorrhage is relatively high in Africa compared with other regions; and the strongest association between hypertension and stroke was also reported in African participants (odds ratio, 4.96 (95% confidence interval, 3.11–7.91) versus 2.79 (95% confidence interval, 1.83–4.75) for developed countries. Factors associated with poor prognosis are impaired consciousness, swallowing problems, incontinence and chest infection based on data from Gambia^48^. Stroke will remain a problem unless urgent action is taken in health promotion and the provision of facilities for optimal care within the resources available. Stroke units can provide crucial support for meeting some of these challenges and are available in some tertiary care facilities^49^.

Dementia is the most common neurodegenerative disease that affects older adults and the oldest old. Data on disease burden is sparse in sub-Saharan Africa because of the relatively young population and the suspected concealment of cases within families^50^. The prevalence in African communities varies between 2.29% and 10.1%, although direct comparison of results between studies may be hampered by differences in the methodology used. The rates are higher in South American countries^51^, in East Asia, the Pacific and South Asia relative to the level of economic development^52^. According to Alzheimer’s Disease International, 58% of those affected live in LMICs, where awareness is considered to be poor^53^. Alzheimer’s is the predominant type of dementia, accounting for between 50% and 80% of cases^15^. Other types are vascular dementia, Lewy body dementia and frontotemporal lobar degeneration.

The risk factors for dementia are similar to those in western countries^43^. An intriguing observation was the lack of association between apolipoprotein E ε4 allele and Alzheimer’s^54^ in sub-Saharan Africa. Furthermore, a recent publication from the Indianapolis-Ibadan group reported significantly lower rates of dementia in Africa compared with ethnically similar populations in the United States^55^. This finding could indicate a paradigm shift in dementia risk and could have a bearing on rising dementia burden as a consequence of changing dietary habits. Vascular factors have also been shown to increase dementia risk. Hypertension after the age of 65 increases the risk of incident dementia in elderly Yoruba Nigerians^56^. A novel presenilin 1 mutation was found to be associated with Alzheimer’s in a South African family and was characterized by early-onset presentation^57^. Other types of dementia associated with neurodegenerative diseases, including frontotemporal dementia, probably exist in LMICs^15^.

Social isolation is linked with cognitive decline owing to limited cognitive stimulation. The extended family system that has so far provided a buffer for the care of older people in LMICs is being eroded by rural–urban migration and economic pursuits^58^. Furthermore, institutionalized care is frowned on in these countries because of the stigma of destitution. Carers face many problems, notably psychological stress and high rates of depression; carer distress, therefore, is an emerging problem. To obviate this, a model of care for LMICs is a creche-type service for those with dementia. Weight loss is another source of concern as this may be a pointer to the development of dementia^59^. Intervention studies in regions where institutionalized care for older people is not an established norm may offer insights for high-income settings.

Dementia is a public health priority and the use of medication is limited in LMICs; consequently, intensive efforts should be directed at identifying the risk factors and making interventions to stem the tide. Education and lifestyle changes (for example, the consumption of healthy diets, physical activity and engagement in community activities to reduce social isolation) can go a long way to preventing dementia. Mild cognitive impairment (MIC) is the intermediate stage between normal ageing and dementia. It is classified into amnestic and non-amnestic types, and each type can affect single or multiple cognitive domains^60^. Single-domain amnestic MCI is a precursor of Alzheimer’s, and it is the most common. The prevalence of MCI varies between 10% and 40% in studies from Africa^15,61–63^. Vascular cognitive impairment follows stroke with white matter lesions and medial temporal atrophy, and manifests as impairment of executive functions^64^. Important risk factors for MCI include age, hypertension, dyslipidaemia, illicit drug use and metabolic disorders^15^. HIV infection also causes mild neurocognitive dysfunction and should be investigated based on clinical judgement.

Parkinson’s disease is the most common movement disorder that affects older people and has both motor and non-motor manifestations. Dementia and depression are common co-morbidities. In Guam, Parkinsonism is associated with Alzheimer’s and amyotrophic lateral sclerosis. Exposure to neurotoxins such as cycasin seems to be important in disease aetiology; oxidative stress also plays a part^65^. Many neurotoxins have been implicated in tropical myelinopathies, including cyanogenic glycosides present in poorly fermented cassava^66^ and β-n-oxalyl-amino-l-alanine present in the chickling pea (*Lathyrus sativus*; also known as the grass pea), which if consumed during drought causes lathyrism^67^. These neurotoxins have not been implicated as risk factors for Parkinson’s disease.

Symptomatic epilepsy in our global ageing population is redefining the epidemiology of a condition previously considered to be a disorder of children and young adults. In older people, the important aetiological factors are stroke, space occupying lesions, metabolic derangements, neurodegeneration and medication side effects. Neurocysticercosis and onchocerciasis are two tropical diseases that cause epilepsy in LMICs and should be looked for and treated. Prescribing cheap, cost-effective medications with minimal side effects and using simplified regimens could reduce the challenge posed by a treatment gap, which leaves most people with epilepsy in resource-limited settings untreated.

## INSIGHTS GAINED FROM WORK IN LMICS

This review of a decade of research into brain disorders in LMICs illustrates the rich diversity of disorders, geographical areas and populations studied. The studies used a variety of methods, approaches and study designs, ranging from qualitative research to clinical or epidemiological approaches, and to translational and basic science projects.

Although each study addresses issues of importance to brain disorders that are relevant to the host country, the body of research also has relevance to high-income populations. Global health research can inform our understanding of the health of all populations and improve health care in high-income settings by exploring the health of key underserved populations, such as recent immigrants or ethnic minorities; enabling research of disorders with high morbidity, but low prevalence, in higher income settings; empowering researchers to challenge existing paradigms; identifying approaches or interventions with better cost-effectiveness than existing standards of care; and identifying best practices with superior outcomes.

## OPPORTUNITIES TO BUILD ON RECENT ADVANCES

A decade of research has established a solid foundation on which to continue to build research collaborations and a body of knowledge about brain disorders in the developing world across the life course. Translating these scientific advances into individual or population-based prevention or treatment interventions that improve brain health is a crucial next step to achieve better health for individuals affected by brain disorders worldwide.

## FUTURE DIRECTIONS

Essentially all research programmes are conducted in a resource-limited setting because of finite budgets, human resources and research infrastructure. Every programme undertaken comes at the cost of the opportunity for another research programme, development activity or investment. As such, some principles for priority setting might be considered. Specific programme content and priorities may vary regionally and development of programmes should be undertaken through partnerships in which LMIC partners direct the content and purpose on the basis of local needs.

The impressive body of work emanating from LMICs and collaborations with researchers from these countries illustrates the value of such international partnerships to all involved (Box 1). Future research needs to continue to address the shared burden of common conditions, including neurodegenerative disorders, stroke and epilepsy (Table 1). For neglected tropical diseases such as cysticercosis, rabies, trypanosomiasis and leprosy, methods developed for the study of rare and orphaned conditions (most of which are neurological) could be applied to these diseases. Although epidemiological and translational research across the spectrum of diseases and methodologies is still needed in resource-limited settings, an increased focus on interventional studies, such as clinical trials or population-based interventions is crucial. Interventions that are culturally relevant and feasible to implement in resource-limited settings, and which can be translated into effective policies that ultimately lead to improved health at both the individual and population levels are the essential next step and need to be prioritized to take full advantage of the body of knowledge generated so far.

## Figures and Tables

**Table 1:** Research priorities to address the neurological burden of disease in low- and middle-income countries

Priorities	Example	Potential approach

Implementation studies of interventions or programmes found to have efficacy or be effective on a small scale	• Screening and treatment for depression and anxiety among people with, or mothers of those with, epilepsy • Evaluate varying systems of dementia care for health, health-related quality of life and cost outcomes	• Scale up of developed programmes with multifaceted, community-based assessments • Randomized studies at a community level
Intersection of neurological NCDs and infectious and/or MCH conditions that remain problematic in LMICs	• Stroke, epilepsy, cognitive impairment in people living with HIV/AIDS • Life-course evaluation of the impact on adult health of childhood malnutrition and obesity	• Accessing populations for study through established HIV services • May require birth cohort, or a comparison of long-term outcomes for countries that did and did not meet Millennial Development Goals
Maximize use of existing data	• Further elucidate the burden of neurological diseases within the framework needed for health-service delivery, treatment and secondary prevention	• Re-framing of GBD 2010 data to quantify relative burden of neurological disorders as manifested rather than as viewed through the root cause • Databases are available that are not focused on neurological health, but which capture relevant exposures and outcomes
Study new disorders that may offer opportunities to gain understanding of mechanisms for neurological disease or injury that are more broadly relevant	• Nodding syndrome and konzo	• Requires collaborations with clinicians, epidemiologists and basic scientists
Clinical trials of potentially affordable interventions informed by insights gained in high-income setting and aimed at improving neurological outcomes	• Avoidance of hyperthermia after neurological injury informed by post-arrest and post-HIE cooling protocols • Possible inclusion of aggressive antipyretics rather than overt cooling	• Pragmatic clinical trials

GBD, global burden of disease; HIE, hypoxic ischemia encephalopathy; LMICs, low- and middle-income countries; MCH, maternal and child health; NCDs, non-communicable diseases

## BOX 1 STUDIES CONDUCTED IN LMICS THAT INFORM RESEARCH IN HIGH-INCOME SETTINGS

- Studies of genetic risk factors for bipolar I disorder have the potential to improve our understanding of these factors in immigrant populations in the United States^68^.
- Proposed studies of stroke prevention in sickle cell disease in Africa^69^ and studies of cognition and/or psychosis in familial populations^70^ in Mexico can directly affect the health of Americans with these disorders.
- Proposed studies of the impact of HIV1 viral diversity on cognition and most studies evaluating the neurological and behavioural outcomes associated with environmental toxins or exposures would not be feasible in the United States, but the results of these studies provide crucial insights into health-related concerns directly applicable to US populations.
- Research on outcomes of guideline-based management of increased intracranial pressure (ICP) rather than invasive ICP monitoring^71^ would not have been possible in the United States given the absence (appropriate or not) of equipoise among clinicians and entrenched practice patterns.
- Resource challenges facing research conducted in LMICs stimulate the development of more cost-effective diagnostics such as the tripolar concentric ring electrode based non-invasive transcutaneous focal stimulation for epilepsy^72^.
